# Effect of postoperative corticosteroids on surgical outcome and aqueous autotaxin following combined cataract and microhook ab interno trabeculotomy

**DOI:** 10.1038/s41598-020-80736-w

**Published:** 2021-01-12

**Authors:** Megumi Honjo, Reiko Yamagishi, Nozomi Igarashi, Chui Yong Ku, Makoto Kurano, Yutaka Yatomi, Koji Igarashi, Makoto Aihara

**Affiliations:** 1 Department of Ophthalmology, Graduate School of Medicine, The University of Tokyo, 7-3-1 Hongo Bunkyo-ku, Tokyo, 1138655 Japan; 2 Southern Specialist Eye Center, Melaka, Malaysia; 3 Department of Clinical Laboratory, The University of Tokyo Hospital, Tokyo, Japan; 4 Department of Clinical Laboratory Medicine, Graduate School of Medicine, The University of Tokyo, Tokyo, Japan; 5 Bioscience Division, Reagent and Development Management, TOSOH Corporation, Kanagawa, Japan

**Keywords:** Experimental models of disease, Outcomes research

## Abstract

To evaluate the effect of postoperative corticosteroids on surgical outcome and autotaxin (ATX) levels after microhook ab interno trabeculotomy combined with cataract surgery (μLOT-CS), prospective, consecutive non-randomized case series comparing outcomes of 30 eyes with primary open angle glaucoma was performed. The aqueous ATX, intraocular pressure (IOP) and glaucoma medications were monitored for 3 months postoperatively. An in-vivo mouse μLOT model was generated. In vitro, ATX and fibrotic changes induced by dexamethasone (Dex) treatment following scratch (S) in cultured human trabecular meshwork (hTM) cells were assessed by immunofluorescence, immunoenzymatic assay, and RT-qPCR. Postoperative ATX at 1 week and the number of antiglaucoma medications at 3 months were significantly lower in non-steroid group, and steroid use was the only variable significantly associated with postoperative medications at 3 months in multiregression analyses. In vitro, ATX activity was significantly upregulated in the Dex + S group, and αSMA was significantly upregulated in the Dex and Dex + S groups. Fibronectin and COL1A1 were significantly upregulated in the S group. μLOT-CS decreased IOP and medications in the overall cohort, and non-use of postoperative steroids resulted in a smaller number of postoperative medications. Limiting postoperative steroids in μLOT may minimize IOP elevation and postoperative fibrosis.

## Introduction

Intraocular pressure (IOP) elevation is hypothesized to result from increased aqueous humor (AH) outflow resistance, mainly in the conventional outflow pathway^1–3^. This resistance involves the trabecular meshwork (TM) and Schlemm’s canal (SC) tissues in cases of primary open angle glaucoma (POAG), which is the most common form of glaucoma^4^. The resistance in the distal pathway due to the damage of collector channels is also implicated in the advanced glaucoma^5^. Cellular responses involving the conventional pathway, such as regulation of the contractile properties of TM cells, remodeling and abnormal accumulation of extracellular matrix (ECM), adhesive interaction, and decreased SC permeability have been associated with glaucomatous degeneration of outflow pathway tissues^6–8^.

Trabeculotomy (LOT) is a surgical procedure in which the TM and inner wall of the SC, the main site of AH outflow resistance, are cleaved. Upon insult, the TM tissue responds by mediating local inflammation and initiating tissue remodeling^9^. We have previously reported that ab externo LOT was effective in reducing IOP, especially when combined with cataract surgery in patients with POAG, steroid-induced glaucoma, and exfoliation glaucoma^10–12^. The recently introduced minimally invasive glaucoma surgery (MIGS) represents a safer and less traumatic surgical intervention for patients with mild to moderate glaucoma, and for those unable to tolerate standard medical therapy^13, 14^. Among the recent MIGS devices and techniques for ab-interno LOT^15–17^, μLOT has proven to be a safe and an effective procedure to lower IOP, with limited postoperative inflammation, especially when combined with cataract surgery (μLOT-CS) in open angle glaucoma (OAG) patients^18–20^.

Concerning postoperative inflammation, the efficacy of phacoemulsification combined with the iStent is comparable to phacoemulsification alone^21^, suggesting that the need for postoperative topical corticosteroid therapy (TCT) after MIGS combined with cataract surgery may be limited^22^. Although TCT has been employed routinely to suppress postoperative inflammation after intraocular surgery, it has been suggested that postoperative TCT may promote IOP elevation^23, 24^. Recently, Salimi et al. compared the surgical outcome of iStent, one of the MIGS for ab-interno LOT, combined with cataract surgery (iStent-CS) with versus without postoperative TCT; they found no significant difference with respect to postoperative inflammation or peripheral anterior synechiae (PAS) formation; however, the steroid group had a higher number of IOP spikes compared with the non-steroid group^25^.

We previously reported that the aqueous level of autotaxin (ATX), an enzyme involved in the generation of lysophosphatidic acid (LPA) via lysoPLD activity, is upregulated in OAG and plays a crucial role in the regulation of TM fibrosis and aqueous outflow resistance^6, 26^. ATX-LPA signaling is involved in multiple biological and pathophysiological processes, including vasculogenesis, pulmonary or liver fibrosis, and tumor progression^27, 28^. We confirmed that ATX is induced by dexamethasone (Dex) treatment in human TM (hTM) cells, and in turn induces a fibrotic response and ECM production in hTM cells via autocrine and paracrine manner^26^.

LPA is a lipid mediator that promotes outflow resistance and mediates IOP elevation. In a previous study, we showed a positive correlation between aqueous upregulation of the ATX-LPA pathway and IOP^29^.

Given that the μLOT-CS procedure is associated with relatively minor trauma to TM tissues, similar to the iStent, we hypothesized that the use of postoperative TCT would provide little benefit in terms of inflammation suppression, but may predispose patients to deleterious postoperative effects such as IOP elevation. In addition, after the μLOT surgical procedure, the remodeling process may begin in the incised TM area. This could alter the profiles of several bioactive factors, including aqueous ATX(especially with corticosteroid steroid treatment), and may play a role in postoperative pathogenic remodeling of outflow pathway tissues.

Here, we conducted a prospective study comparing the outcomes of POAG patients who underwent μLOT-CS with versus without postoperative TCT, and explored surgical outcomes according to postoperative TCT and aqueous ATX use. Aqueous ATX was upregulated in a mouse μLOT model. In addition, we demonstrated that Dex treatment induced significant ATX expression, as well as fibrotic and cytoskeletal changes, in hTM cells when combined with scratch (S) as a mechanical stress in vitro.

## Results

### Demographic data and surgical outcome of μLOT-CS in the steroid and non-steroid groups

The demographic and baseline clinical characteristics, and preoperative and postoperative values, of each group are presented in Table 1. There were no significant differences in age, sex, preoperative IOP, number of antiglaucoma medications, mean Mean deviation (MD), or preoperative aqueous ATX level. All eye surgeries (30 eyes; 15 each in the steroid and non-steroid groups) were performed without any complications.

**Table 1:** Demographic characteristics and preoperative and postoperative values.

Variables	Steroid	Non-steroid	*P* value
Patients (n)	15	15	
Number of eyes (n)	15	15	
**Gender**			
(Male: female)	6:9	7:8	NS*
**Age (years)**			
Mean ± SD	70.7 ± 9.4	75.4 ± 6.7	NS**
Range	50–84	63–87	
**Pre IOP (mmHg)**			
Mean ± SD	14.7 ± 3.7	15.7 ± 3.3	NS**
Range	10–20	12–24	
**Post IOP (1 W) (mmHg)**			
Mean ± SD	15.9 ± 5.0	13.0 ± 3.2	NS (*P* = 0.0627)**
Range	11–25	9–21	
**Post IOP (3 M) (mmHg)**			
Mean ± SD	13.7 ± 2.8	12.6 ± 2.0	NS**
Range	10–20	10–18	
**Mean deviation (dB)**			
Mean ± SD	− 9.7 ± 4.0	− 8.3 ± 3.9	NS**
Range	− 3.82 to − 14.45	− 2.09 to − 15.31	
**SUN cell score (1 W)**			
Mean ± SD	1.1 ± 0.6	1.1 ± 0.5	NS**
Range	0–2	0–2	
**SUN flare score (1 W)**			
Mean ± SD	1.0 ± 0.4	1.1 ± 0.5	NS**
Range	0–2	0–2	
**Pre medication**			
Mean ± SD	2.5 ± 1.1	1.9 ± 1.1	NS**
Range	1–4	1–4	
**Post medication (3 M)**			
Mean ± SD	1.6 ± 1.3	0.1 ± 0.4	0.0002**
Range	0–4	0–1	
**Pre ATX**			
Mean ± SD	850.5 ± 659.7	625.9 ± 264.8	NS**
Range	454.8–1742.1	246.9–1095.1	
**Post ATX (1 W)**			
Mean ± SD	869.8 ± 285.1	616.6 ± 174.4	*P* = 0.0066**
Range	556.0–1460.8	336.7–1049.5	
IOP spike	6	2	NS*
Highest IOP	20.9 ± 7.3	15.7 ± 5.7	*P* = 0.0148**
Range	11–30	10–35	

*Pre* preoperative; *Post* postoperative; *1 W* 1 week; *SD* standard deviation; *IOP* intraocular pressure; *ATX* autotaxin. IOP spike: > 25 mmHg.

*Fisher's exact test, **Mann–Whitney *U* test.

**Figure 1:**
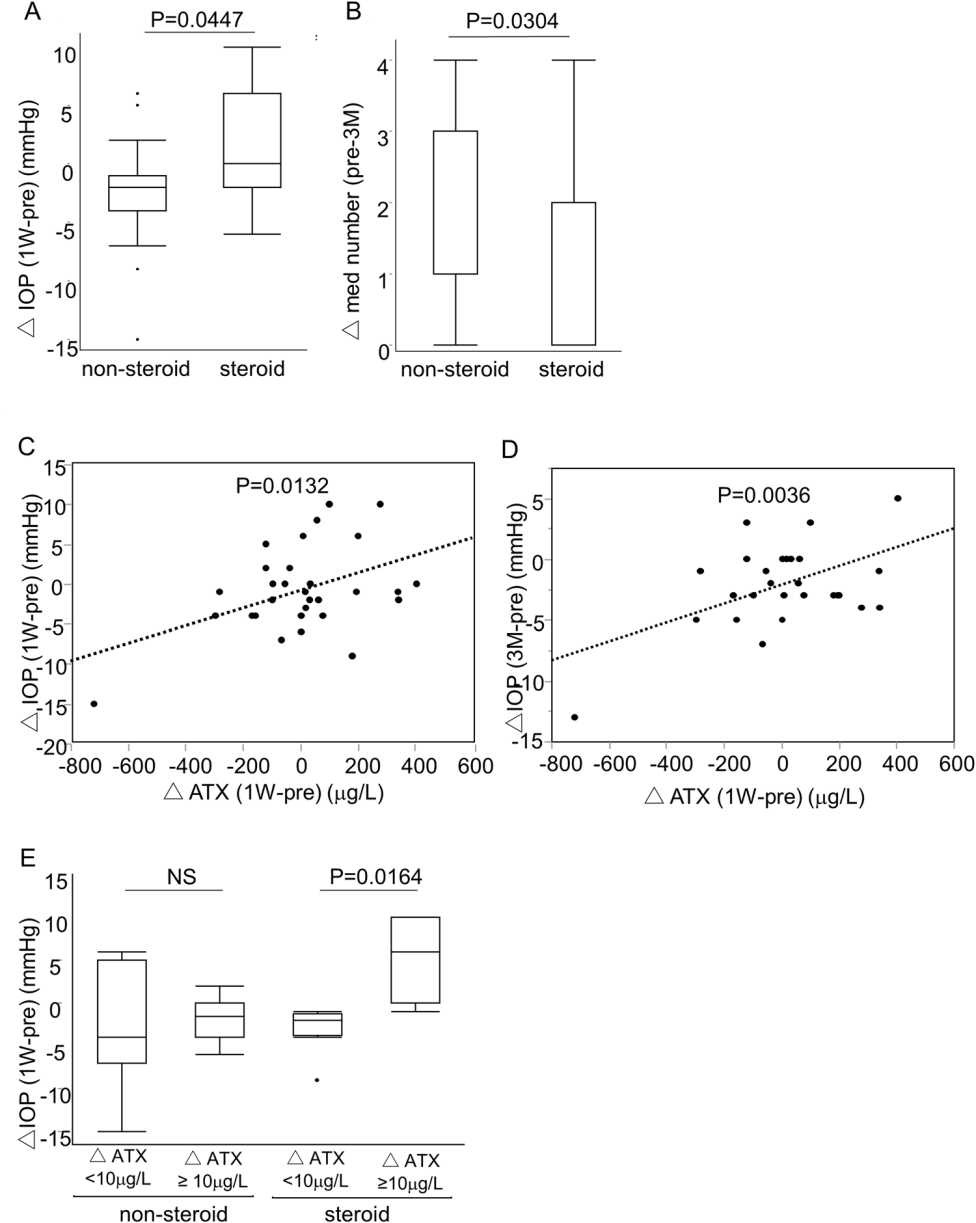
Relationship between the amount of change in IOP and ATX levels after μLOT-CS in the non-steroid and steroid groups. (**A**) Intraocular pressure (IOP) reduction at 1 week compared with preoperative IOP; change in IOP levels (△IOP; 1 W − pre) in the non-steroid and steroid groups. (**B**) Decrease in the number of glaucoma medications at 3 months (△med number; pre − 3 M) compared with the preoperative number of medications in the non-steroid and steroid groups. (**C**) Correlation between △IOP (1 W − pre) and the change in autotaxin (ATX) (△ATX; 1 W − pre). D. Correlation between IOP decrease (preoperative level vs. 3-month level; △IOP; 3 M − pre) and the △ATX (1 W − pre). E. △IOP (1 W − pre) in the non-steroid and steroid groups: comparison between groups in which the ATX level was increased by ≥ 10 µg/L compared with the preoperative level. μLOT-CS: microhook ab interno trabeculotomy combined with cataract surgery.

Overall, the IOP and number of antiglaucoma medications decreased significantly, from 15.2 ± 3.5 mmHg and 2.2 ± 1.1 medications preoperatively to 13.1 ± 2.5 mmHg and 0.9 ± 1.2 medications at 3 months postoperatively (*P* = 0.0008 and *P* < 0.0001, respectively). The IOP was significantly lower at 1 week postoperatively in the non-steroid group (*P* = 0.0306, Wilcoxon signed-rank sum test), while there was no significant IOP reduction in the steroid group (*P* = 0.8137). Figure 1A illustrates the significant difference between the two groups in IOP decrease at 1 week compared with the preoperative IOP level (△IOP; 1 W − pre); there was a decrease in IOP in the non-steroid group at 1 week when antiglaucoma medications other than pilocarpine had not yet been added to the regimen. At 3 months postoperatively, although there was no significant difference in IOP between the two groups, the number of antiglaucoma medications was significantly lower in the non-steroid group (*P* = 0.0002) (Table 1). The decrease in the overall number of medications was also significantly greater in the non-steroid group (*P* = 0.0304) (Fig. 1B).

There were no severe postoperative complications in either group. No eyes exhibited hyphema with a height of more than 0.5 mm, or the need for additional washout or postoperative intervention. No eyes showed prolonged inflammation or a significant decrease in corneal endothelial cell density, and visual acuity was improved in all cases in both groups (data not shown). There was no significant difference in the extent of postoperative inflammation between the two groups, and only a few eyes showed an AC cell or flare grade of 1 or higher at 1 week, according to the Standardization of Uveitis Nomenclature (SUN) working group criteria (Table 1)^30^. After 3 weeks, AC cell or flare grades were less than one in all eyes. Although few IOP spikes occurred during the first week postoperatively, spikes were nevertheless observed in six eyes in the steroid group (40%) and two eyes in the non-steroid group (13.3%). The mean highest IOP value was significantly larger in the steroid group (*P* = 0.0148) (Table 1). The highest IOP value typically occurred during the first week postoperatively.

### Relationship between aqueous ATX level and surgical outcome in the steroid and non-steroid groups

In the overall cohort, there was no significant difference in the mean aqueous ATX level between the preoperative value and that at 1 week postoperatively. Although there was no significant difference in the preoperative aqueous ATX level between the two groups, the ATX level at 1 week postoperatively was significantly lower in the non-steroid group (Table 1). One-way repeated measures ANOVA revealed that the change in ATX level was significantly greater in the non-steroid group (*P* = 0.0163).

Figure 1C,D illustrates the significant positive correlation between the change in ATX (preoperative vs. 1 week) and △IOP (1 W − pre) (*P* = 0.0132), which persisted at 3 months postoperatively (*P* = 0.0036) even though the anti-glaucomatous medications had been added at that time. This suggests a possible relationship between the aqueous ATX level and IOP regulation. The aqueous ATX level at 1 week increased by ≥ 10 µg/L compared with the preoperative level in 15 eyes (50%) in the overall cohort, while the rate of increased ATX was almost the same in both groups (8 and 7 eyes in the non-steroid and steroid groups, respectively). The increased ATX level did not correlate with the △IOP in the non-steroid group; however, a significant correlation was observed in the steroid group (*P* = 0.0164, Fig. 1E). We speculated that the steroid-induced increase in the ATX level may have played a role in IOP elevation seen in some subjects of the steroid group.

To determine the factors associated with the number of postoperative medications at 3 months after adjusting for demographic and clinical variables, we performed simple and multiple regression analyses (Table 2). In the simple regression analysis, the highest IOP, steroid use, preoperative and postoperative (1-week) ATX levels, postoperative (1-week) IOP, and number of preoperative medications were positively associated with the number of postoperative medications; age, sex, and pre- and postoperative (3-month) IOP levels were not statistically significant. In the multiple regression analysis, steroid use was the only variable significantly associated with the number of medications used at 3 months.

**Table 2:** Regression analysis of factors associated with the number of postoperative medications.

	Simple regression analysis					Multiple regression analysis				
	B	95% CI		*P* value	B	B	95% CI		β	*P* value
**Post med**										
Age	− 2.212	− 0.607	0.050	0.089						
Sex	0.066	− 0.489	0.216	0.409						
Steroid-use	0.265	0.340	0.803	0.0002	(adjusted R2 = 0.4553)	0.9393	0.1463	1.7322	0.3995	0.022
Pre med	0.360	0.035	0.658	0.033		0.1152	− 0.2871	0.5174	0.1064	0.559
Pre ATX	120.14	0.088	0.687	0.016		0.0002	− 0.0017	0.0021	0.0487	0.850
1 W ATX	121.73	0.234	0.759	0.0017		0.0005	− 0.0018	0.0028	0.1070	0.672
Pre IOP	0.457	− 0.217	0.488	0.499						
highest IOP	3.405	0.291	0.784	0.0006		0.0588	− 0.03	0.148	0.34	0.19
Post 1 W IOP	1.613	0.100	0.694	0.0138		− 0.002	− 0.132	0.1288	− 0.006	0.98
Post 3 M IOP	0.616	− 0.0712	0.593	0.112						

B = regression coefficient or partial regression coefficient.

95% CI = 95% confidence interval of partial regression coefficient.

β = standardized partial regression coefficient.

adjusted R2 = adjusted coefficient of multiple determination.

### LysoPLD activity in the mouse model of μLOT

**Figure 2:**
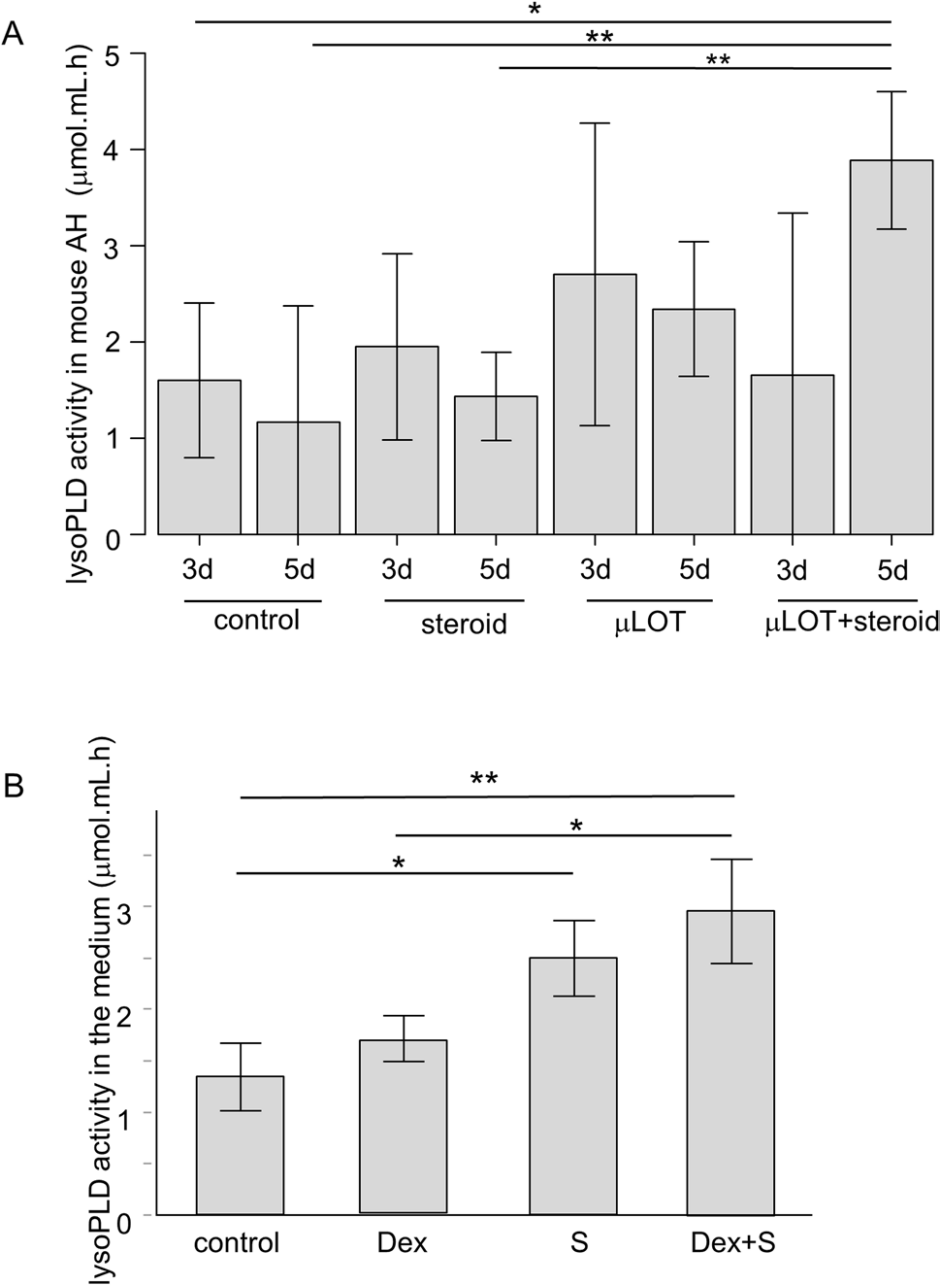
Increased ATX activity in the mouse model of μLOT and conditioned medium of hTM cells. (**A**) LysoPLD activity in the aqueous humor (AH) of the mouse model of μLOT. LysoPLD activity was significantly elevated in the μLOT + steroid group at 5 days after surgery compared with the control and steroid groups. (**B**) LysoPLD activity in the conditioned medium. LysoPLD activity was significantly elevated in the mechanical stress (S) group compared with the control, and in the dexamethasone + stress (Dex + S) group compared with the control and Dex groups. *P < 0.05, **P < 0.01. Data are representative of three independent experiments. hTM: human trabecular meshwork.

In clinical studies, we could not rule out effects of preoperative medication, or variation in IOP or aqueous ATX levels among individual subjects. Thus, we examined the effects of μLOT and simultaneous steroid treatment on aqueous ATX levels using the μLOT mouse model. Figure 2A illustrates the significant increase in lysoPLD activity in the μLOT + steroid group at 5 days compared with the control and steroid-only groups.

### lysoPLD activity in the conditioned medium

LysoPLD activity was significantly higher in the conditioned medium of cultured hTM cells in the S and Dex + S groups compared with the Dex group (Fig. 2B).

### Effects of Dex and S on ATX expression and the fibrotic response in cultured hTM cells

**Figure 3:**
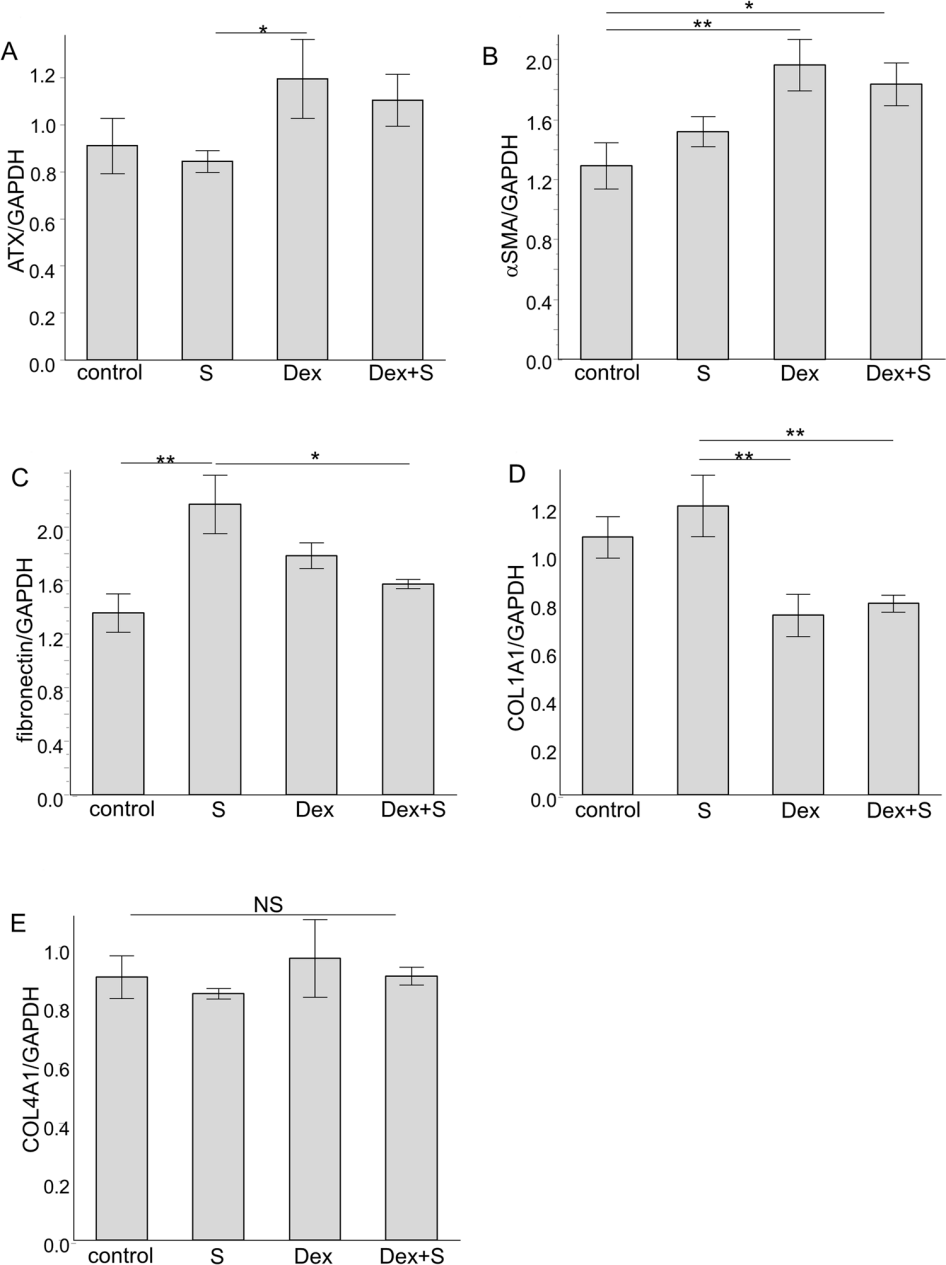
Expression levels of ATX and fibrotic markers in hTM cells. (**A**) The relative mRNA expression of ATX was significantly higher in the Dex group. (**B**) Alpha smooth muscle actin (αSMA) was significantly higher in the Dex and Dex + S groups compared with the control. (**C**) Fibronectin was significantly upregulated in the S group, and the upregulation was suppressed by Dex treatment. (**D**) COL1A1 was higher in the S group but lower in the Dex and Dex + S groups. (**E**) COL4A1 showed no significant changes. RT-qPCR was performed with GAPDH primers as an internal control for input DNA. Data are the average values of four independent DNA samples from treated cells. *P < 0.05; **P < 0.01; hTM: human trabecular meshwork.

We analyzed the mRNA expression levels of ATX and fibrotic markers in Dex and S-exposed hTM cells using RT-qPCR (Fig. 3). ATX expression was significantly increased in the Dex group, but not in the S or Dex + S groups (Fig. 3A). αSMA was significantly upregulated in the Dex and Dex + S groups, but not in the S group (Fig. 3B). Interestingly, fibronectin was significantly upregulated in the S group, but this upregulation was suppressed by Dex treatment (Fig. 3C). Regarding COL1A1 and CoL4A1, no significant upregulation was observed in the Dex or S treatment groups, but it was seen in the Dex + S group (Fig. 3D,E).

We also evaluated the relative mRNA expressions of TGF-β1-3 (Supplemental Fig. 1). The expression of TGF- β1 was significantly upregulated in the S and Dex + S groups. Also, there existed significant difference between Dex and S. The expression of TGF-β2 showed no significant difference among groups. The expression of TGF- β3 was significantly upregulated in S.

### Expression of ATX and fibrotic markers in hTM as assessed by immunocytochemistry and western blotting

**Figure 4:**
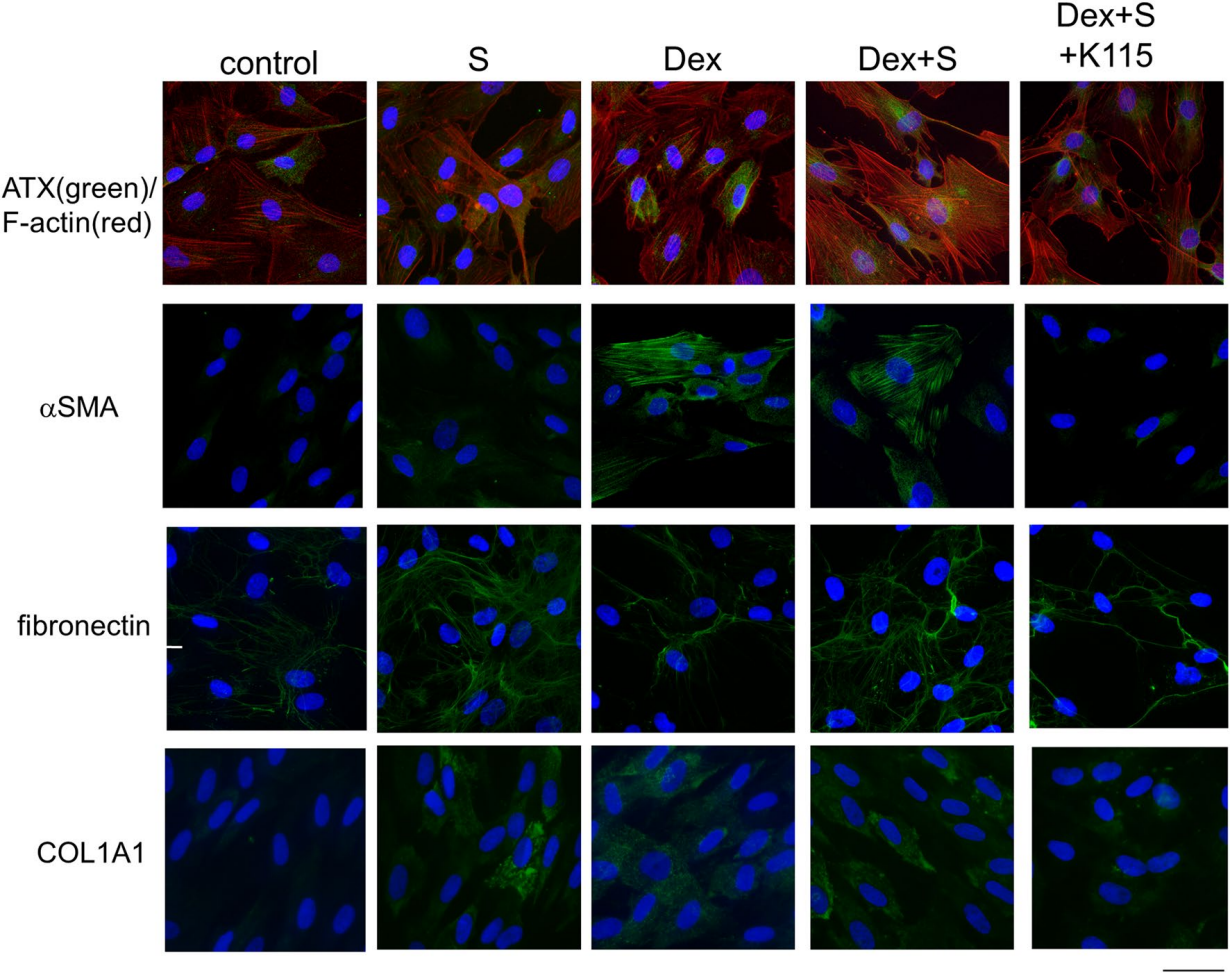
Immunostaining showing cellular responses in hTM cells. Immunostaining for ATX (green), nuclei/DNA (4′,6-diamidino-2-phenylindole DAPI; blue) and F-actin (red) (upper panels) was performed in hTM cells treated with Dex (100 nM), Dex and S, or Dex + S + K115 for 24 h. Immunostaining for αSMA (green, second upper panels), fibronectin (green, third upper panels), and COL1A1 (green) (lower panels) was also conducted. Cell nuclei were counterstained with DAPI (blue). Bars, 50 μm.

**Figure 5:**
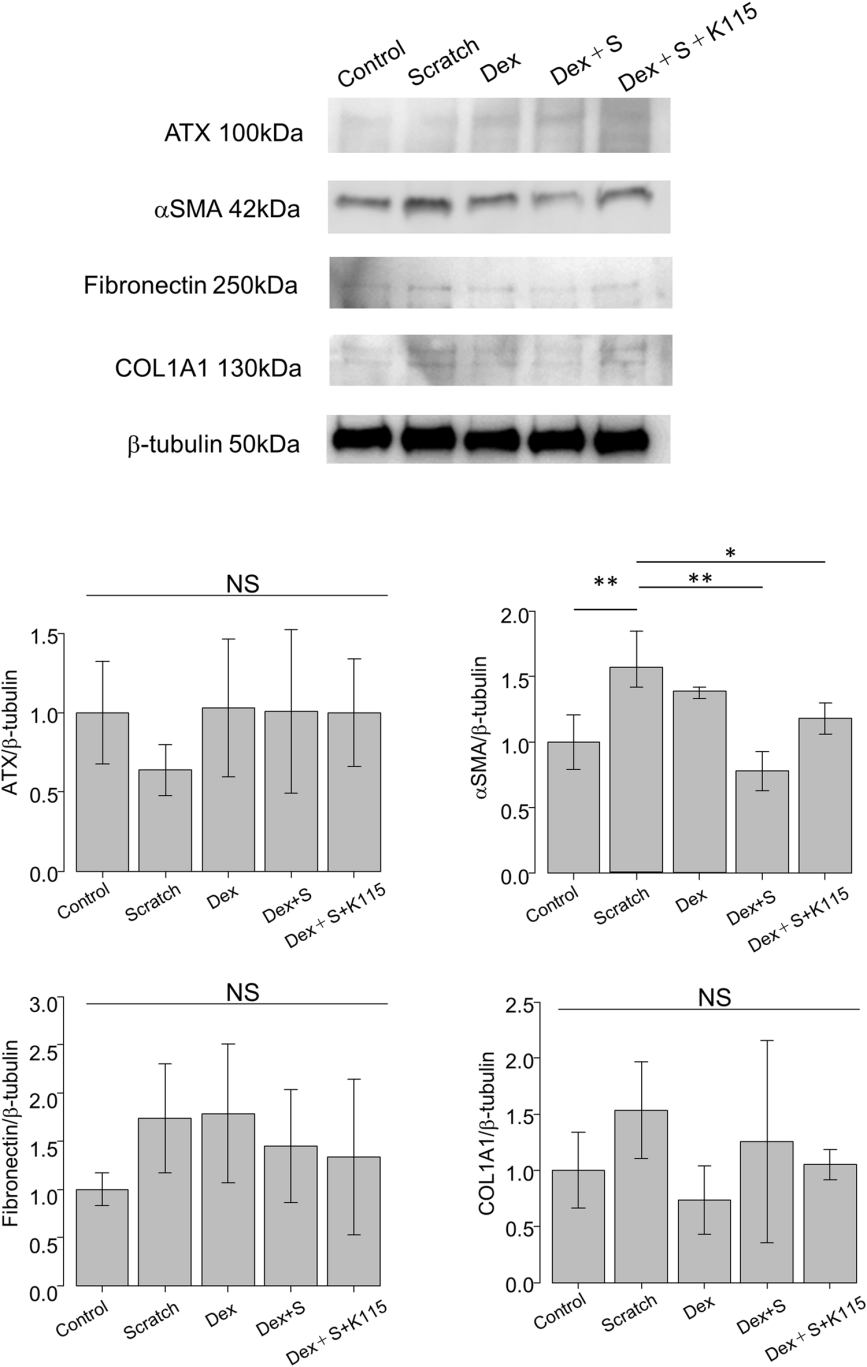
Western blotting of ATX and fibrotic markers in hTM cells. Western blotting of ATX, aSMA, fibronectin and COL1A1 in hTM cells. The representative bands are shown in (**A**), and the relative expression of ATX, aSMA, fibronectin and COL1A1 compared to the loading control (β-tubulin) are shown in (**B**) (n = 3). *P < 0.05, **P < 0.01.

Immunocytochemistry (Fig. 4) and western blotting (Fig. 5) was used to examine the protein expression of ATX and cytoskeletal and fibrotic markers. A ROCK inhibitor (K115) was used to determine whether the cellular response could be suppressed, given that the Rho-ROCK pathway is downstream of ATX-LPA signaling. Figure 4 illustrates the significant upregulation in the expression of ATX (green) in the Dex, and Dex + S groups, although the RT-qPCR results suggested significant upregulation of ATX only in the Dex group. However, this result is in good accord with the lysoPLD activity shown in Fig. 2B. Although it showed the similar tendency, western blot analysis failed the significant difference of ATX expressions among groups. F-actin was also upregulated in the S and Dex groups, and enhanced in the Dex + S group in immunohistochemistry. Regarding fibrotic markers, αSMA was significantly upregulated in the Dex and Dex + S groups in immunocytochemistry (Fig. 4) and in S group and in western blot analysis (Fig. 5), in accordance with the RT-qPCR findings. In immunohistochemical analysis, fibronectin and COL1A1 upregulation were more obvious in the S group compared with the Dex and Dex + S groups. However, it showed the similar tendency, but these upregulations of cytoskeletal and fibrotic markers and attenuation by K115 were not significant by quantitative analysis using western blotting.

## Discussion

Topical corticosteroids are effective in reducing anterior segment inflammation. The vulnerability of ocular tissue to damage and inflammation has warranted routine use of TCT after intraocular interventions. However, these medications are also known to be associated with several side effects, including delayed wound healing, lowered resistance to infections, cataract formation, and increased IOP^31^. Postoperative TCT (and resultant high corticosteroid concentrations in ocular tissue, especially in the conventional aqueous outflow pathway) has been suggested to result in increased ECM deposition, reorganization of the cytoskeleton, fibrosis, and decreased TM cell phagocytosis, leading to increased aqueous outflow resistance and IOP elevation^24^. This may be especially problematic for glaucoma patients; indeed, in more than 90% of POAG patients receiving corticosteroid treatment, IOP increases^32–34^. For postoperative management after intraocular interventions in glaucoma patients, it is critical not only to minimize inflammation, but also to suppress and control IOP elevation.

In the present study, we assessed the impact of postoperative TCT on surgical outcome and aqueous ATX levels after μLOT-CS. In this prospective consecutive case series of POAG patients, a significant postoperative reduction in IOP was observed and fewer glaucoma medications were required. There was no significant difference in postoperative inflammation or IOP at 3 months according to the presence or absence of TCT in the postoperative regimen. However, significant reductions were observed in the postoperative IOP level and variation in aqueous ATX at 1 week, and in the number of antiglaucoma medications at 3 months, in the non-steroid group compared with the steroid group; steroid use was the only variable significantly associated with the number of postoperative (at 3 months) medications in multiregression analysis results. This suggests that TCT treatment after μLOT-CS can lead to an increase in the aqueous outflow resistance and IOP, thus necessitating a larger number of glaucoma medications.

Initially, we focused on the IOP within the first week postoperatively, i.e., when no glaucoma medications other than pilocarpine had been started. △IOP (1 W − pre) was significantly larger in the non-steroid group (Fig. 1A), and the highest IOP was significantly higher in the steroid group (Table 1). As shown in Fig. 1C, △IOP (1 W − pre) showed a significant correlation with the change in aqueous ATX level; this correlation persisted at 3 months (Fig. 1D), suggesting that the postoperative change in ATX level could continue to influence postoperative IOP control. The ATX level at 1 week postoperatively may vary not only according to postoperative TCT, but also in accordance with inflammation severity and residual hemorrhage. Indeed, half of the eyes in each group showed an elevated (by > 10 µg/L) ATX level, and there was no significant group difference in the proportion of such subjects. However, interestingly, a significant correlation between △IOP (1 W − pre) and elevated ATX was seen only in the steroid group (Fig. 1E). In a similar comparative study on the surgical outcome of iStent-CS with versus without postoperative TCT by Salimi et al., in which surgical trauma to TM tissue was even less extensive compared with μLOT-CS, IOP spike frequency was higher in the steroid group^25^. They observed the clinical outcome postoperatively for 6 months and there were no difference in number of postoperative medications during that period, however the number of postoperative medications was lower in non-steroid group at postoperative 3 months (0.62 ± 1.00 in steroid group and 0.27 ± 0.67 in nonsteroid group), although the difference was not significant (*P* = 0.071). Based on these results, we speculate that steroid-induced ATX elevation or variation may be related to postoperative fibrotic changes in TM tissue, and the resultant increases in aqueous outflow resistance and IOP.

Trauma from cataract surgery is known to produce a cascade of inflammatory pathways due to the release of arachidonic acid and production of prostaglandins via cyclooxygenase (COX)-1 and -2 enzyme activation. Excessive prostaglandin release likely leads to breakdown of the blood-aqueous barrier, possibly resulting in clinical symptoms such as pain, hyperemia, light sensitivity, and vision-threatening cystoid macular edema^23^. Although TCT has long been the gold standard treatment modality for controlling this inflammation, by blocking the arachidonic pathway and preventing prostaglandin formation, several clinical studies have demonstrated that postoperative NSAIDs may act synergistically with the steroid by blocking COX enzymes, and could be superior to steroid treatment when used alone^35, 36^. Regarding the potential superiority of TCT over NSAID, sufficient evidence is lacking at this point, even after uncomplicated phacoemulsification^37^. Several studies have examined the benefits of TCT after MIGS combined with cataract surgery. In a previous μLOT-CS case series with routine postoperative TCT, a significant IOP reduction was seen, from a preoperative mean of 16.4 ± 2.9 mmHg (slightly higher than in our study) to 11.8 ± 4.5 mmHg postoperatively; the results also showed a drop in the number of medications required, from 2.4 to 2.1 at 9.5 months postoperatively^19^. Notably, the IOP reduction in this earlier study was comparable to that in the steroid group in the current study^19^. In the same study, the postoperative AC flare was 6.3 pc/ms, which is within range of the baseline level. In another study, it was reported that the postoperative AC flare after μLOT returned to baseline at 1 month and was significantly lower compared with that associated with trabeculectomy at 6 months^38^. In the present study, we did not monitor the patients for AC cell or flare quantitatively, so additional study using lase flare-cell meter will be needed for quantitative analysis^39^; however, there were no significant differences in postoperative inflammation graded by SUN working group criteria between the non-steroid and steroid groups, and none of the eyes in either group showed prolonged inflammation at 1 week requiring additional anti-inflammatory medications. It is necessary to monitor postoperative inflammation closely and to adequately control prolonged iritis if it occurs. Given that μLOT may be accompanied by postoperative hyphema or breakdown of the blood-aqueous barrier, the results of the present study suggest that NSAIDs alone may be sufficient, and could potentially replace steroids in the treatment of typical inflammation after μLOT-CS. However, at the same time, we have to pay attention to the extent of TM incised in the present study, which was limited in the nasal for 4 o′clock. Different extents of LOT can result in more inflammation when the angle is opened to a greater extent. Further study will be needed to elucidate that NSAIDs alone can attain more severe inflammation.

Concerning the influence of postoperative hyphema and the breakdown of the blood-aqueous barrier on ATX expression, we previously found no significant correlation between the level of ATX in peripheral blood and AH^29^. However, we also noted that oral administration of steroids decreased the circulating level of ATX, which is mainly secreted from adipose tissue under physiological conditions^40^. Concerning the increase in serum concentration of steroids in the systemic circulation, TCT for 1 week postoperatively may have limited effects; however, further study of this issue is needed. Although our clinical results suggested a possible relationship between TCT and elevated aqueous ATX levels, variations in preoperative medications, baseline IOP, disease duration, ATX levels, and other clinical variables may have biased the results. Therefore, we used the in-vivo mouse model of μLOT to determine whether the aqueous ATX level increased when S delivered to the TM was combined with TCT. We found that ATX activity was significantly upregulated in mouse eyes at 5 days after μLOT with TCT. Additionally, there was a significant difference between μLOT-only and μLOT with TCT, suggesting a role of steroids in the upregulation of ATX in the in-vivo mouse model (Fig. 2A).

After confirming that the aqueous ATX was increased by the μLOT plus TCT regimen in animal studies, we examined the expression levels of ATX, and cytoskeletal and fibrotic proteins induced by Dex and S, in cultured hTM in vitro.

In treated hTM cells, we confirmed that ATX activity, protein expression levels, and the cytoskeletal response were upregulated by Dex and S, and further enhanced by combining the Dex and S treatments, as shown in Figs. 2B and 4. Regarding fibrotic markers, αSMA expression was not significantly elevated in the S group; however, mRNA and protein expression levels were significantly increased in the Dex and Dex + S groups (Figs. 3B and 4). It has been postulated that sustained activation of Rho promotes the transdifferentiation of hTM cells into myofibroblasts^41, 42^. As we have reported previously, ATX upregulated by Dex treatment could trigger Dex- and Dex + S-induced myofibroblast-like transitions in hTM cells^26^. In contrast, the expression levels of fibronectin and COL1A1 were significantly increased only in the S group; additional Dex treatment suppressed upregulation of these fibrotic markers (Figs. 3C,D, and 4). An increase in fibronectin in the aqueous outflow pathway in cases of steroid-induced glaucoma and POAG, and in TGF β2, has been suggested to play a key role in the regulation of fibronectin synthesis^43^. The cellular response may also vary according to the involvement of S. TGFβ2 is a strong fibrotic mediator that is also upregulated by steroids^44^. Although we did not observe the significant upregulation of TGFb2 in hTM cells by S or Dex + S (Supplemental Fig. 1), it may be attributable that the period of Dex treatment was relatively short in the present study. Further study will be needed to understand the biological processes involved. Taken together, the results of our in-vitro and animal studies suggest enhanced cellular and fibrotic responses of hTM cells when S is combined with steroid treatment. However, further study will be needed to determine whether steroid treatment may be effective in suppressing fibrosis upon the onset of this condition.

The results of this study are instructive for surgeons regarding the potential impact of TCT for μLOT-CS; it appears that TCT should be minimized or avoided after μLOT-CS due to concerns regarding myofibrogenic changes in hTM cells and resultant fibrosis of TM tissue. In the present study, we opened TM in the nasal for 4 o′clock in all patients. Tanito et al., reported that the different extents of LOT did not affect IOP reduction or postoperative medications^19^, so we performed the minimized extent of LOT with the uniformity in all patients. However, TCT may be effective when severe inflammation occurs, presumably in cases when the greater extent of TM is incised.

There were several limitations to the present study. First, due to its prospective nature and limited number of recruited patients, aqueous ATX levels varied among the groups, although there was no significant group difference in preoperative values. In addition, the present study was a non-randomized study. Although we performed experiment using mouse model of μLOT to confirm the upregulation of aqueous ATX after μLOT, further randomized clinical study will be required. Second, the follow-up period was short. Our institution functions as a university hospital performing operations and patients usually receive postoperative medical care at community medical organization after 3 months, therefore we did not continue the observation after 3 months although there was no deposition of patients. Further studies with a larger number of patients and a longer follow-up will be needed. Third, we could not fully elucidate an additive effect between scratch and Dex treatment on the activity of ATX-ROCK axis in vitro. The responsiveness varied depending on each hTM cells, so we would like to perform further study of ELISA using patient’s aqueous humor or TM cells collected during the MIGS surgery to clarify this issue in the future study. Finally, we did not evaluate the formation of cross-linked actin networks, or upregulation of TGFβ2, myocilin, or other factors reportedly associated with POAG and steroid-induced glaucoma^45^. Other than ATX, other cytokines could be affected by TCT and may also influence TM scarring, as we observed the upregulation of TGFb1 and TGFb3 in hTM cells by S (Supplemental Fig. 1). Additional studies comparing the levels of ATX and other factors including multiplex assay at various time points could further elucidate the mechanisms underlying TM tissue after surgery.

In summary, to our knowledge, this is the first study to report that postoperative TCT may be related to a higher number of postoperative glaucoma medications, possibly due to increased aqueous ATX and TM tissue fibrosis after μLOT-CS, and presumably after MIGS as a stand-alone procedure as well. The need for postoperative TCT, and its association with a higher number of glaucoma medications, should be assessed in larger, multicenter randomized control cohort studies including patients with a wider range of glaucoma subtypes and baseline IOP values, and longer follow-up periods.

## Materials and methods

### Patients and clinical evaluation

A prospective, consecutive non-randomized case series comparing the outcomes of 30 eyes of 30 Japanese POAG patients who underwent μLOT-CS, with versus without postoperative TCT, was conducted at the University of Tokyo Hospital. Thirty patients who underwent μLOT-CS between January 2019 and June 2019 to control IOP and treat visually relevant cataracts were enrolled in this study. All participants required surgical treatment for medical reasons and were using at least one antiglaucoma medication. The study was approved by the Institutional Review Board of the University of Tokyo and was registered with the University Hospital Medical Information Network Clinical Trials Registry of Japan (Registration ID: UMIN000027137, 26/04/2017). All procedures conformed to the Declaration of Helsinki. Written informed consent was obtained from all patients.

All subjects underwent a comprehensive ophthalmic examination, including a review of medical records, best-corrected visual acuity (BCVA), slit-lamp biomicroscopy, Goldmann applanation tonometry, gonioscopy, dilated fundus examination, and visual field examinations. The inclusion criteria were adult POAG patients (> 20 years old) who had undergone μLOT-CS with a minimum follow-up of 3 months. Patients with a history of ocular conditions other than OAG, steroid-induced glaucoma, cloudy corneas likely to impair gonioscopic examination, prior eye surgery (including laser trabeculoplasty), prior trauma, ocular surface disease, topical use of steroids within 3 months postoperatively, or a history of systemic use of steroids were excluded. The maximum preoperative IOP was determined within 1 month prior to surgery.

All surgeries were performed by one surgeon (M.H.). All the patients who received μLOT-CS by M.H. during the period, agreed to enter the study and met criteria were enrolled in the study consecutively. Participants were numbered according to when they entered the study, with “1” representing the first participant. The steroid group included subjects 1–15, who received TCT (0.1% betamethasone sodium phosphate four times a day) for 1 week postoperatively and took an antibiotic for 3 days preoperatively and 1 week postoperatively, a topical nonsteroidal anti-inflammatory drug (NSAID; Nepafenac 0.1% three times a day) for 8 weeks, and 1% pilocarpine (three times a day) for 4 weeks postoperatively. All patients taken off postoperative pilocarpine after 4 weeks. Subjects 16–30 were classified into the non-steroid group and received the same regimen but without TCT.

Postoperative evaluations were conducted at 1 day, 1 week, 3 weeks, 6 weeks, and 3 months postoperatively. Every evaluation visit included slit-lamp biomicroscopy, Goldmann applanation tonometry IOP measurement, and scoring of the number of antiglaucoma medications (in the case of combined medications, the sum of the active agents was used). AC cell or flare was graded according to the SUN working group criteria^30^. Throughout the study, all examinations were conducted by two glaucoma specialists (M.H. and M.A.). A postoperative IOP spike was defined as an IOP increase ≥ 25 mmHg. Postoperative glaucoma medications were adjusted as follows. For IOP spikes occurring during the first week postoperatively, 250 mg of oral acetazolamide was prescribed as a single dose, as needed. After 1 week, topical antiglaucoma medications were added when the IOP increased by 3 mmHg or more with respect to baseline.

### Surgical technique of μLOT-CS and AH collection

μLOT-CS was performed as previously reported^9^. In brief, AH samples were collected and stored at the beginning of cataract surgery after paracentesis had been performed^29^. Following a routine procedure of phacoemulsification and IOL implantation, with the anterior chamber (AC) filled with a viscoelastic material, the AC angle was visualized using a Swan–Jacob lens (Ocular Instruments, Bellevue, WA, USA). A Tanito *ab interno* microhook (Inami & Co, Ltd., Tokyo, Japan) was inserted through the side port, and the inner wall of the TM at the nasal 120° (from 2 to 6 o′clock in the right eye and from 6 to 10 o′clock in the left eye) was incised. The viscoelastic material was removed through I/A using balanced salt solution (BSS; Alcon Laboratories, Inc., Tokyo, Japan).

At 1 week postoperatively, AH was collected in the outpatient clinic again. Under topical anesthesia, approximately 70–100 μL AH was obtained using a 30-gauge syringe at the slit lamp and collected into a 1.5 mL PROTEOSAVE SS tube (Sumitomo Bakelite, Tokyo, Japan), which was stored at − 80 °C until sample processing.

### Assessment of ATX and lysoPLD activity

The ATX levels in human AH was determined via a two-site immunoenzymatic assay with an ATX assay reagent using the Tosoh AIA system (Tosoh, Tokyo, Japan). ATX activity in the mouse AH and conditioned medium of cultured TM cells was evaluated based on lysoPLD activity, as described previously^29^.

### Mouse model of μLOT

All animals used in this study were treated in accordance with the ARVO Statement for the Use of Animals in Ophthalmic and Vision Research and the dictates of the Animal Use Committee of the University of Tokyo. Male C57BL/6 J mice were purchased from Japan Tokyo Laboratory Animals Science Co. Ltd. (Tokyo, Japan). Mice were bred and housed in clear cages loosely covered with air filters. The temperature was maintained at 21 °C under a 12-h:12-h light: dark cycle. All mice had access to food and water ad libitum. We used mice older than 8 weeks of age. It is well known that the conventional outflow tissue in mouse eyes is very similar to human eyes in terms of anatomy and physiology^46^, and the reported diameter of SC visualized by OCT in living mouse was approximately 150–200 μm^47, 48^. In the mouse model of μLOT, a microneedle made of borosilicate glass which was an optimal size for mice eyes (tip diameter: 75–100 μm; outer diameter: 1.0 mm; World Precision Instruments, Sarasota, FL, USA) was inserted into the AC from the paracentesis and the angle area was scratched for 120° width. Antibiotic drug treatment, with or without 0.1% betamethasone eye drops, was then applied for 5 days.

#### Preparation of hTM cell culture and treatment

Primary hTM cells were isolated from human donor eyes and characterized as described previously according to the recommendation by Keller et al.^49^ Only well-characterized normal hTM cells, in which Dex-induced myocilin upregulation was confirmed by real-time quantitative polymerase chain reaction (RT-qPCR; passages 3–8), were used for subsequent studies. Three independent cell lines from different donors were used and the reproducibility was confirmed for every cell lines. All in-vitro experiments were performed after overnight serum starvation. Cells were treated simultaneously with the indicated concentrations of ROCK inhibitor (K115, 10 µM; provided from Kowa Company, Nagoya, Japan) and Dex stimulation (100 nM; Sigma-Aldrich, St. Louis, MO, USA), with or without mechanical scratch (S) for 24 h or the appropriate vehicle control, and with the addition of the indicated concentration of fetal bovine serum. The S was in the form of four scratches of each well of the hTM cell monolayer, made with the blunt tip of an 18-G needle.

### Immunocytochemistry

Immunocytochemistry was performed as described previously^26, 50^. The primary antibodies were anti-alpha smooth muscle actin (αSMA; (Dako-Agilent, Santa Clara, CA, USA), anti-fibronectin (Santa Cruz Biotechnology, Dallas, TX, USA), rhodamine phalloidin (Thermo Fisher Scientific, Waltham, MA, USA) and anti-ATX (MBL, Nagoya, Japan). Alexa Fluor 488 secondary antibodies were purchased from Thermo Fisher Scientific.

### RNA extraction and RT-qPCR

To measure ATX levels in hTM cells and assess ECM gene expression , RT-qPCR was performed as described previously^26, 50^. Serum-starved (for 24 h) confluent cultures of hTM cells were treated with Dex (100 nM), with or without S, for 24 h and then subjected to RT-qPCR. mRNA levels were quantified by the ΔΔCt method. The sequences of PCR primers used were as follows: GAPDH: forward 5′-GAGTCAACGGATTTGGTCGT-3′ and reverse 5′-TTGATTTTGGAGGGATCTCG-3′; ATX: forward 5′-ACAACGAGGAGAGCTGCAAT-3′ and reverse 5′-AGAAGTCCAGGCTGGTGAGA-3′; fibronectin: forward 5′-AAACCAATTCTTGGAGCAGG-3′ and reverse 5′-CCATAAAGGGCAACCAAGAG-3′; COL1A1: forward 5′-CAGCCGCTTCACCTACAGC-3′ and reverse 5′-TTTTGTATTCAATCACTGTCTTG-3′; COL4A1: forward 5′-TAGAGAGGAGCGAGATGTTC-3′ and reverse 5′-GTGACATTAGCTGAGTCAGG-3′; and αSMA: forward 5′-CCGACCGAATGCAGAAGGA-3′ and reverse 5′-ACAGAGTATTTGCGCTCCGAA-3; TGF-β1, forward, 5′- CCCAGCATCTGCAAAGCTC-3′ and reverse 5′- GTCAATGTACAGCTGCCGCA; TGF-β2, forward, 5′- TGCCGCCCTTCTTCCCCTC-3′ and reverse 5′- GGAGCACAAGCTGCCCACTGA-3′; TGF- β3, forward, 5′- GGTTTTCCGCTTCAATGTGT, and reverse 5′-TATAGCGCTGTTTGGCAATG. Target gene expression was normalized to that of GAPDH mRNA. All tests were conducted in triplicate to ensure reproducibility.

### Western blotting

To measure ATX levels in hTM cells and assess ECM gene expression, western blotting was performed as described previously^26, 50^. After 1 dpi, cells were collected in RIPA Buffer (Thermo Fisher Scientific) containing protease inhibitors (Roche Diagnostics, Basel, Switzerland), sonicated, and centrifuged. The following protein concentration measurement and SDS-PAGE was performed as previously described.55 Protein bands were transferred to PVDF membranes (Bio-Rad Laboratories) and the membranes were immersed in Tris-buffered saline with Tween 20 (TBST) containing primary antibody. After washing, the membranes were immersed in TBST containing secondary antibody and reacted with ECL substrate (Thermo Fisher Scientific). Protein bands were detected by ImageQuant LAS 4000 mini (GE Healthcare, Chicago, IL, USA). Primary antibodies were: anti-ENPP2 (1:1000; Abcam, Cambridge, MA, USA), anti-aSMA (Sigma-Aldrich Co., LLC St. Louis, MO USA, 1:1000), anti-fibronectin (1:1000; Abcam, Cambridge, MA, USA), anti-collagen type I (1:1000; Cell Signaling Technology, Danvers, MA, USA). HRP-conjugated second antibody (1:10,000) was purchased from Thermo Fisher Scientific (Waltham, MA USA). b-tubulin served as the loading control. All membranes were stripped of antibodies using WB Stripping solution and incubated with mouse monoclonal antibody b -tubulin (1:1000), and subsequently with H goat anti-mouse IgG antibody (1:2000). Densitometry of scanned films was performed using ImageJ 1.49 (NIH Bethesda, MD, USA), and results are expressed relative to the loading control (b -tubulin).

### Statistical analysis

Statistical analyses were performed using JMP Pro 15 software (SAS Institute, Inc., Cary, NC, USA). The preoperative and postoperative clinical values of each group were compared using a Wilcoxon signed-rank sum test. Other parameters were analyzed using the Mann–Whitney U test, Chi-square test, or Fisher's exact test. One-way repeated measures analysis of variance (ANOVA) was used to compare the groups across repeated measurements. The independent effects of the IOP, ATX level, and patient characteristics on the number of postoperative ATX medications at 3 months were evaluated using simple and multiple linear regression analyses. Variance inflation factors were calculated to check for multicollinearity. Group differences in experimental results were analyzed using one-way ANOVA with Tukey’s post hoc test. All in vitro experimental data are shown as the mean ± standard deviation, unless otherwise noted. All data represent the means of at least three independent experiments. A *P* value < 0.05 was considered to indicate statistical significance.

## Supplementary Information

Supplementary Information 1.

Supplementary Information 2.
