# Supplementary material for: Effect of postoperative corticosteroids on surgical outcome and aqueous autotaxin following combined cataract and microhook ab interno trabeculotomy
Source: Sci Rep. 2021 Jan 12;11:747. doi: 10.1038/s41598-020-80736-w (PMC7804433; doi:10.1038/s41598-020-80736-w)
Supplement: Supplementary file 1 — Supplementary Information 1. [file 41598_2020_80736_MOESM1_ESM.docx]

**Supplemental Figure 1. Expression levels of TGFb1, 2 and 3 in hTM cells**

The expression of TGFb1 was significantly upregulated in S and Dex+S. There existed significant difference between Dex and S. The expression of TGFb2 showed no significant difference among groups. The expression of TGFb3 was significantly upregulated in S. Data are the average values of four independent DNA samples from treated cells. *P < 0.05, **P < 0.01

**Supplemental Figure 2.** **Uncropped gel images of western blotting of ATX, fibronectin, COL1A1, aSMA and β-tubulin.**

Uncropped gel images of western blotting of ATX (A, 100kDa), fibronectin (B, 250kDa), COL1A1 (B, 130kDa), αSMA (C, 42kDa) and β-tubulin (D, 50kDa) in hTM cells shown in Figure 5.
