# Supplementary material for: Effect of postoperative corticosteroids on surgical outcome and aqueous autotaxin following combined cataract and microhook ab interno trabeculotomy
Source: Sci Rep. 2021 Jan 12;11:747. doi: 10.1038/s41598-020-80736-w (PMC7804433; doi:10.1038/s41598-020-80736-w)
Supplement: Supplementary file 2 — Supplementary Information 2. [file 41598_2020_80736_MOESM2_ESM.pptx]

## Slide 1
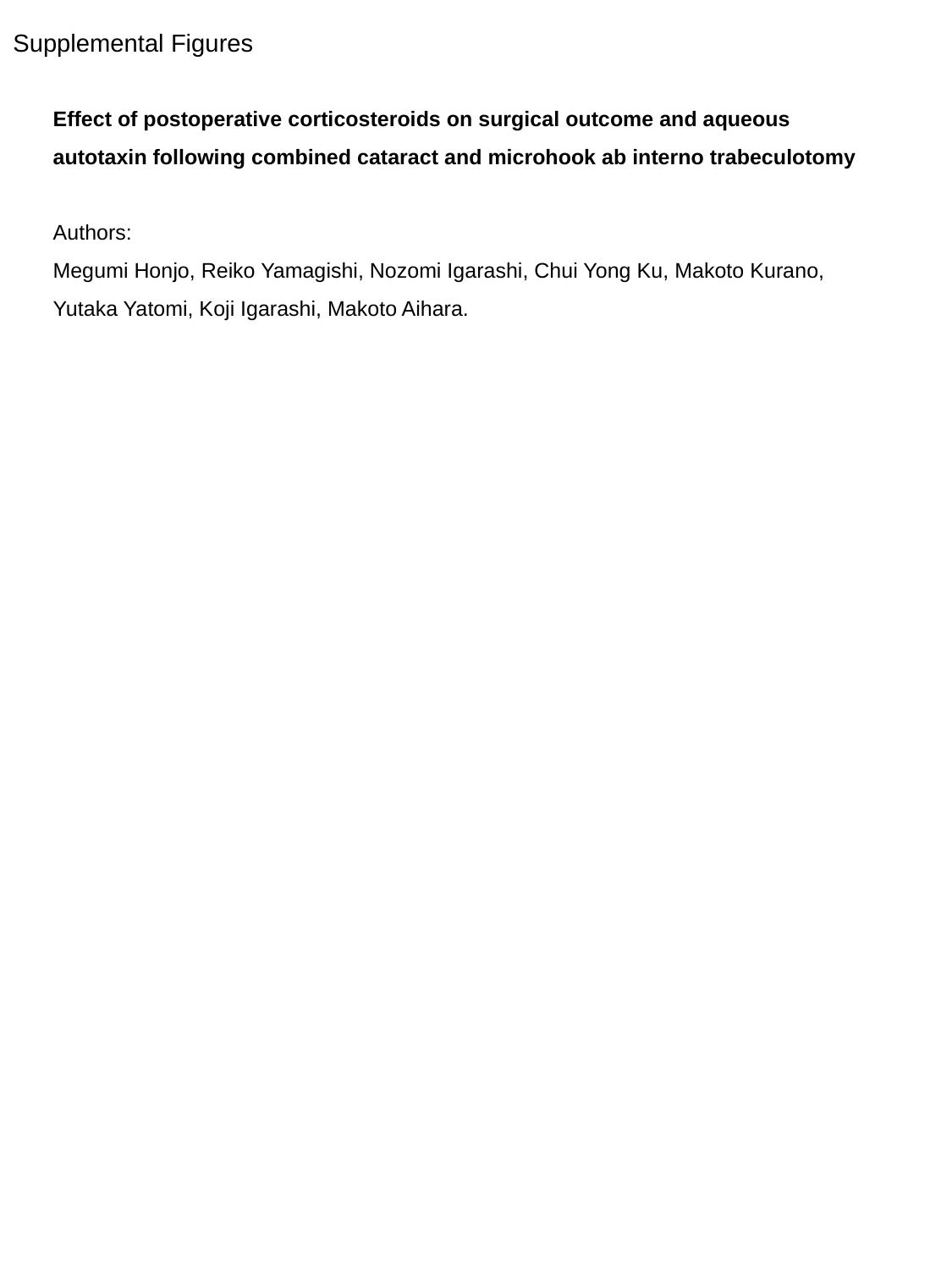

Supplemental Figures
Effect of postoperative corticosteroids on surgical outcome and aqueous autotaxin following combined cataract and microhook ab interno trabeculotomy
Authors:
Megumi Honjo, Reiko Yamagishi, Nozomi Igarashi, Chui Yong Ku, Makoto Kurano, Yutaka Yatomi, Koji Igarashi, Makoto Aihara.

## Slide 2
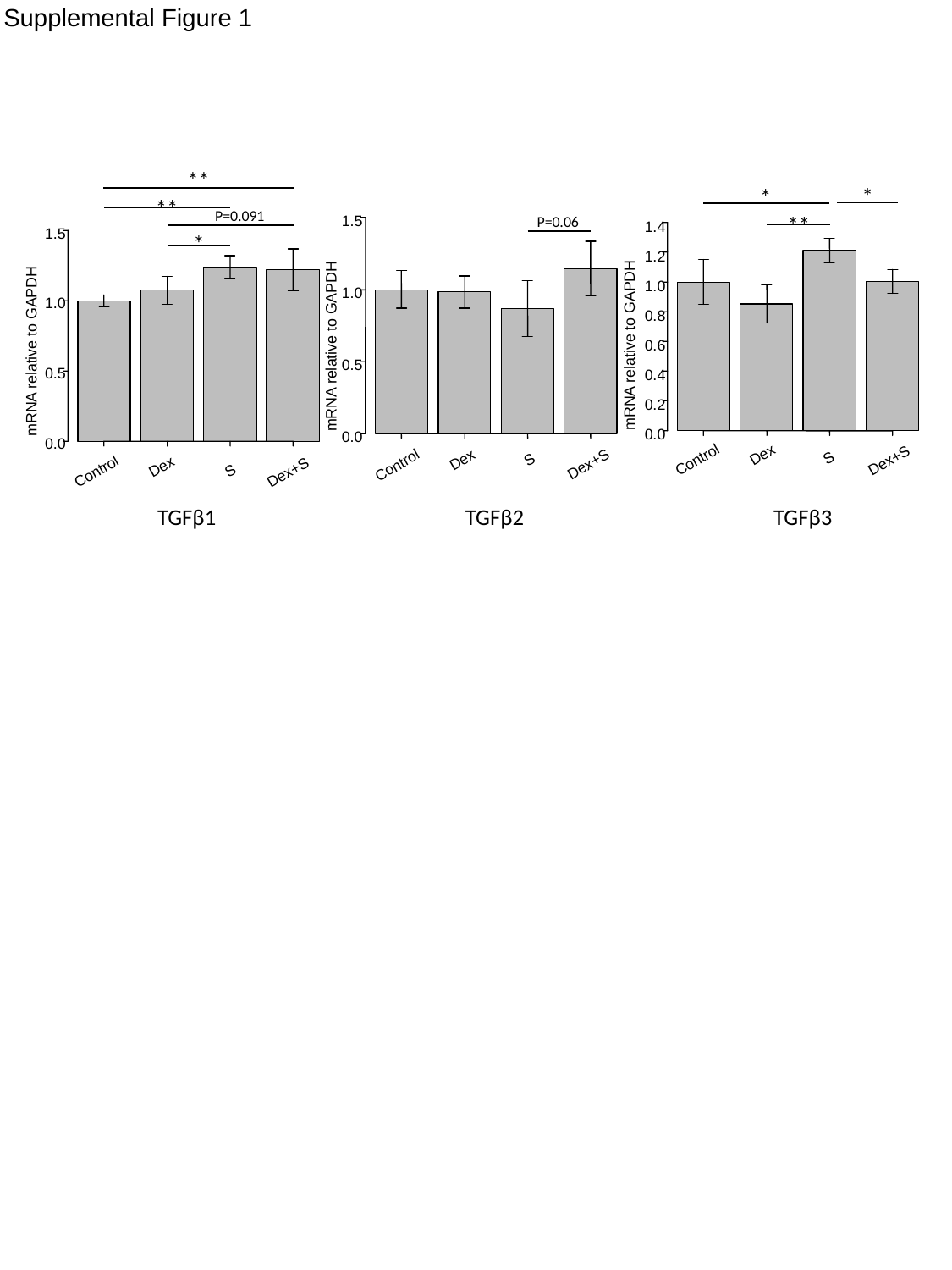

Supplemental Figure 1
**
**
P=0.091
*
1.5
1.0
mRNA relative to GAPDH
0.5
0.0
Dex
S
Control
Dex+S
*
*
**
1.4
1.2
1.0
0.8
0.6
mRNA relative to GAPDH
0.4
0.2
0.0
Dex
S
Control
Dex+S
P=0.06
1.5
1.0
mRNA relative to GAPDH
0.5
0.0
S
Dex
Dex+S
Control
TGFβ2
TGFβ3
TGFβ1

## Slide 3
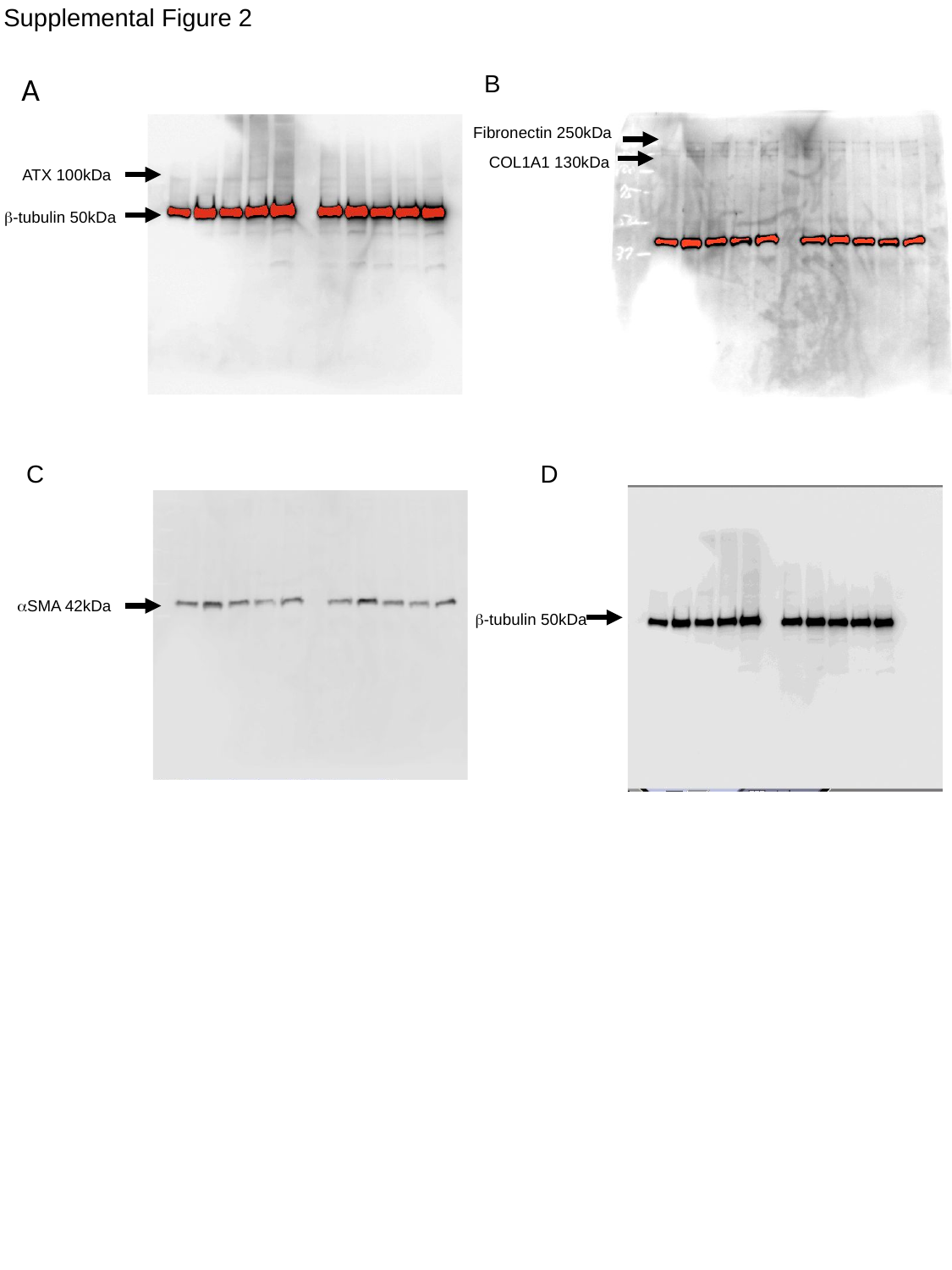

Supplemental Figure 2
B
A
Fibronectin 250kDa
COL1A1 130kDa
ATX 100kDa
b-tubulin 50kDa
C
D
aSMA 42kDa
b-tubulin 50kDa
